# Recent advances in electronic skins: material progress and applications

**DOI:** 10.3389/fbioe.2022.1083579

**Published:** 2022-12-14

**Authors:** Hua-Li Cao, Sui-Qing Cai

**Affiliations:** Department of Dermatology, Second Affiliated Hospital of Zhejiang University School of Medicine, Hangzhou, China

**Keywords:** electronic skins, health monitoring, material, stretchability, self-healing, human-machine interface

## Abstract

Electronic skins are currently in huge demand for health monitoring platforms and personalized medicine applications. To ensure safe monitoring for long-term periods, high-performance electronic skins that are softly interfaced with biological tissues are required. Stretchability, self-healing behavior, and biocompatibility of the materials will ensure the future application of electronic skins in biomedical engineering. This mini-review highlights recent advances in mechanically active materials and structural designs for electronic skins, which have been used successfully in these contexts. Firstly, the structural and biomechanical characteristics of biological skins are described and compared with those of artificial electronic skins. Thereafter, a wide variety of processing techniques for stretchable materials are reviewed, including geometric engineering and acquiring intrinsic stretchability. Then, different types of self-healing materials and their applications in electronic skins are critically assessed and compared. Finally, the mini-review is concluded with a discussion on remaining challenges and future opportunities for materials and biomedical research.

## Introduction

Developing a skin bioelectronic system requires the consideration of many aspects, including material and structure selection and device properties. Nowadays, new flexible electronic materials, such as intrinsically conductive polymers (Tee and Ouyang, 2018), nanomaterials (Jung et al., 2022), and carbon-based polymeric materials (Wang et al., 2018b), are being explored to enhance skin bioelectronic systems (Sanderson, 2021). The tissue-like materials for skin bioelectronics require the following capabilities (Oh and Bao, 2019): 1) stretchability to strains generated by various dynamic deformations caused by movement, 2) self-healing ability to extend its lifetime when mechanical failure occurs, and 3) biocompatibility for long-term stability. The human skin can be used as a reference for flexible/stretchable electronic devices that stick seamlessly to human skin. Artificial skin-like materials have been developed for flexible electronics based on the characteristics of human skin (Gao et al., 2016). To deal with the mismatched behaviors at the interfaces of tissue-like materials and skin tissues, a soft-hard interaction framework has been proposed to formulate new soft-hard interfaces (Fang et al., 2022). This framework will contribute to the future improvement of soft-hard interfaces in living composites or engineered tissues with diverse stimuli-responsive or adaptable behaviors. Here, we present several biomechanical aspects of human skin and the recent progress of materials for skin bioelectronic systems. Finally, the remaining challenges and opportunities are summarized.

## Biomechanical aspects of skin

### Components of skin tissues

**FIGURE 1:**
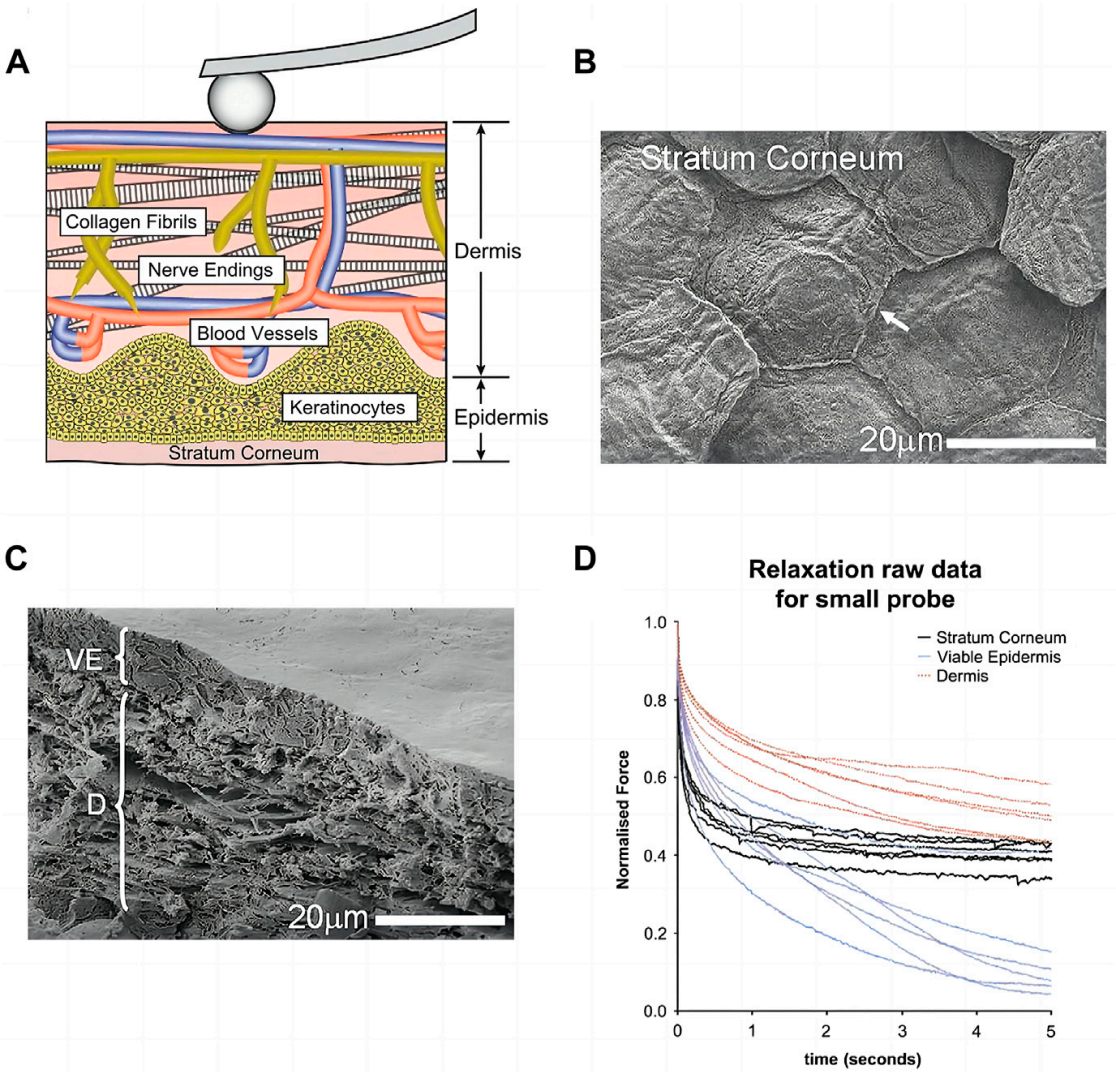
Biomechanical aspects of skin **(A)** Schematic of dermis: cells, blood vessels, nerves, and randomly oriented collagen fibril network within the dermal matrix. Reproduced with permission from ref (Oftadeh et al., 2018). Copyright 2018 Elsevier **(B)** The stratum corneum (SC) of the skin showing plate-like corneocytes (arrow) viewed by CryoSEM **(C)** the viable epidermis (VE) and Dermis (D) **(D)** Data from force relaxation experiments showed skin layers exhibit clear viscoelastic behavior with an initial fast drop in force followed by a clear plateau. **(B–D)** Reproduced with permission from ref (Crichton et al., 2011). Copyright 2011 Elsevier.

As the body’s outermost layer, human skin requires strong pliability to maintain its extensibility, elasticity, tensile strength and firmness. There are mainly three layers of the skin: the outer stratum corneum (SC) layer composed of dead keratinocytes (10–20 μm thick), the inner viable epidermis (VE) layer formed from living cells (30–110 μm thick) (Kendall et al., 2007), and the underlying dermis that gives tensile resistance to the skin (1–2 mm thick). The skin comprises multiple cell types and surrounding extracellular matrix (Palmquist et al., 2022). Cells in the skin are surrounded by extracellular matrix (ECM) components, including collagen, fibronectin, laminin, elastin, proteoglycans, tenascin, and microfibrils. As shown in Figure 1A, cells not only receive signals from the surrounding ECM but also actively remodel it through the secretion of cell-derived ECM. Conversely, the ECM provides certain biomechanical features and regulates cell phenotype, attachment, migration, and proliferation in human skin. Human dermal fibroblasts (HDF) mechanical responses are affected by the stiffness of the ECM. When exposed to stiffer substrates and a more stretched and organized actin cytoskeleton, cells experience a higher elastic modulus in their plasma membrane. Moreover, the cells grow faster, straighter, and migrate slower on stiffer than on soft substrates. Under mechanical forces arising intrinsically, cells of skin reshaped tissue architecture by cell-cell adhesion through the cytoskeleton and interaction with the ECM (Aragona et al., 2020).

### Mechanical properties in skin tissues

Skin tissues exhibit complex rate- and time-dependent mechanical properties (Fontenele and Bouklas, 2022), resulting in “trainable behaviors.” They can be caused by viscoelasticity, poroelasticity (Oftadeh et al., 2018), or other anisotropic and nonlinear strain-stress relationships from the skin tissue (Figures 1B–D). Skin tissues are highly dynamic and viscoelastic with both instantaneous elastic and time-dependent viscous behaviors (Crichton et al., 2011). Young’s modulus varies significantly throughout the skin strata, with the quasi-static variation in mechanical Young’s modulus of the viable epidermis and dermis to be 2.9–11.1 MPa and 18–100 MPa respectively (Silver et al., 2021). The dynamic parameters of mouse skin dermis tested by compressive nanoindentation using 5 μm probe tip radius are as follows: non-fibrillar elastic modulus $E_{m}=2.9\pm 1.3kPa$ , fibrillar modulus $E_{f}=27.7\pm 6.7kPa$ , hydraulic permeability $k=\left(1.47e\pm 0.23\right)10^{-13}m^{4}/Ns$ , viscoelastic modulus $E_{V}=2.1\pm 0.85kPa$ , relaxation time T=21.1±8.6ms (Oftadeh et al., 2018). Each parameter could affect the frequency response: low and high frequency moduli are affected by $E_{m}$ and $E_{f}$ , respectively; the frequency location of the peaks in the poro- and viscoelastic phase angle is affected by k and T ; the magnitude of the dynamic modulus at the viscoelastic peak in phase is affected by $E_{v}$ .

Elastic modulus tested by micro-indentation of individual skin strata were strain rate-dependent (Meliga et al., 2017). The initial relaxation of the dermis, stratum corneum and viable epidermis showed quick relaxation followed by a long plateau, and quantitatively dermis retained the greatest mechanical stiffness (Crichton et al., 2011). High collagen (about 80%–90% type I and 10%–20% type Ⅲ, with elastic modulus near 4.4 GPa) and elastin (around 4 MPa) content may contribute to the mechanical stiffness of dermis (Silver et al., 2001). Besides, skin has high biomechano-robustness resulting from fracture energy of 30000 J/m^2^ (Davis et al., 2004) and excellent self-healing ability to bear deformation and tear. The layer fracture energies for the SC, VE, and dermis were 70 pJ μm^−2^, 2 pJ μm^−2^ and 12 pJ μm^−2^, respectively, tested by micropenetrators using a layered finite-element model (Meliga et al., 2017).The viscoelasticity of the ECM results from the breakdown and reformation of weak cross-links, the disentangling of entanglements, and the unfolding of proteins. Noncovalent bonds are the majority of cross-links in the ECM network that can slide, break, or slip. This allows energy to be dissipated and creep and stress relaxation to occur. Polymer disentanglement and unfolding of ECM proteins also dissipates energy and makes the ECM viscoelastic. Therefore, skin-inspired viscoelastic materials should obtain the energy dissipation features combined with elastic solids and visous fluid. As water flows into or out of the cells and ECM, poroelasticity of the tissue occurs. When ECM is compressed, hydraulic pressure will increase, and water will flow away from it; the resulting ECM poroelasticity causes energy dissipation (Kosheleva et al., 2022). The less permeable cell membrane leads to a different mechanism in cell poroelasticity. Additionally, cytoskeleton deformation can also result in poroelastic effect.

## Need for advanced tissue-like materials

Despite the versatility of synthetic chemistry, traditional materials still cannot achieve certain combinations of viscoelasticity and poroelasticity. In biological tissues, these combinations frequently occur, which are needed for applications such as wearable electronics, tissue engineering and soft robotics. With tremendous mechanical mismatch, silicon’s modulus is a thousand times greater than the skin’s. Therefore, composite materials with structures similar to cell-ECMs may exhibit nonlinear and dynamic characteristics similar to those of tissues (Shim et al., 2021; Wang C. et al., 2022). To enhance their performance and stability, tissue-like materials are usually composed of hydrogels, polymers, and nanomaterials.

Because of their similar mechanical properties to tissues, hydrogels have been widely used as biomaterials (Hu et al., 2022; Jiang and Song, 2022). For example, by incorporating starch granules into hydrogel, Fang et al. fabricated tissue-like materials (Fang et al., 2020). Mechanical stress on an ECM-like hydrogel matrix induced shakeup and training dynamics of the incorporated starch grains. The dynamic behaviors enabled the tissue-like properties of programmability, anisotropy, and trainability. Bai et al. (2022) developed a mucosa-like conformal hydrogel coating. By first absorbing micelles, then forming covalent interlinks with polymer substrate through interface-initiated hydrogel polymerization, a thin hydrogel layer similar to the epithelial layer was achieved. Covalent interlinks mimicking lamina propria formed help the outer hydrogel layer (mimicking the epithelial layer) abut the underlying substrate (mimicking the muscularis mucosae) tightly. The hydrogel coating exhibited mucosa-matched Young’s modulus of 1.1 ± 0.1 kPa, prominent aqueous lubrication (coefficients of friction = 0.018 ± 0.003), and robust interfacial bonding with the substrate against peeling (peeling strength = 1218.0 ± 187.9 J m^−2^). This mucosa-like conformal hydrogel coating can protect the mucosa from invasive stimuli. Liang et al. engineered a hydrogel with isotropic fatigue resistance, with a fatigue threshold over 1,500 J m^−2^ in two arbitrary directions (Liang et al., 2022). Inspired by the structure–mechanics relationship of heart valves, which could reversibly open and close over 3000 000 000 cycles in a 75-year lifetime, they developed the bidirectional freeze-casting strategy for fabricating lamellar microstructures of soft materials with isotropically extreme properties to resist crack propagation. The tensile curves of this 2D poly (vinyl alcohol)/graphene oxide hydrogel along two orthogonal in-plane directions was identical, and there was no obvious crack propagation of the hydrogel sample under an energy release rate of 1520 J m^−2^ along any in-plane directions (with a pre-cut notch ≈1/5 of the over width) over 30000 cycles of stretching.

The recently reported tissue-like materials above may provide alternatives to conventional soft materials in the applications of next-generation electronic skins.

## Tissue-like deformable skin electronics

Highly personalized electrical, thermal, mechanical, and biochemical signals constantly released from our body, indicating our emotions, health, and actions. Electrophysiological signals are produced by electrical activity in the heart, brain, and muscles, for instance. Skin electronics are considered ideal platforms to collect the bio-signals of human skin (Wang Y. et al., 2022) and display information for monitoring of health (Someya et al., 2016; Wang et al., 2018a; Zhang et al., 2022) because of their important characteristics, such as excellent stretchability, fast self-healing capability, and great skin conformability.

**FIGURE 2:**
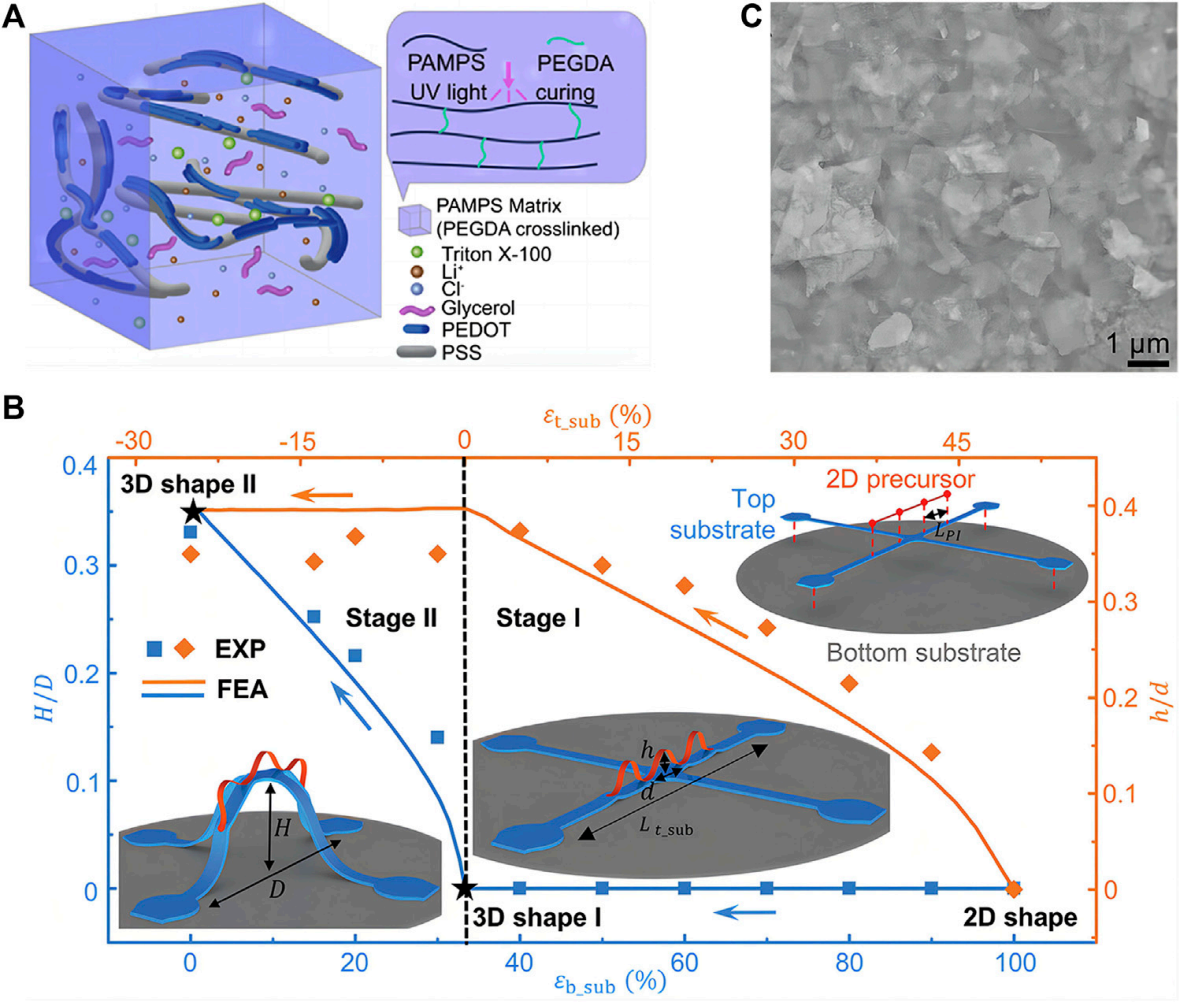
Advanced tissue-like materials for skin electronics **(A)** The schematic diagram of the polymer-hydrogel EEG electrode matrix. Polyethylene glycol diacrylate was used as a cross-linker for the formation of hydrogel. Reproduced with permission from ref (Hsieh et al., 2022). Copyright 2022 Elsevier **(B)** A representative set of 3D mesostructures formed by mechanically guided hierarchical assembly and dependence of their geometries on various design parameters. Reproduced with permission from ref (Zhao et al., 2022). Copyright 2022 Wiley **(C)** SEM images showing VDWTFs assembled from staggered 2D nanosheets. Scale bar, 1 μm. Reproduced with permission from ref (Yan et al., 2022). Copyright 2022 AAAS.

Using stretchable electronics and advanced physiological measurements, Rogers and co-workers developed the devices for neonatal intensive care (Chung et al., 2019). After on-board analysis, the devices provided long-term and continuous real-time monitoring of skin temperature, photoplethysmograms, and ECGs, which yielded measurements of a range of physiological parameters. Wang and co-workers designed a polymer-hydrogel EEG electrode with long-term stability and a lower electrode-skin interfacial impedance value than gel-based electrodes. This electrode was applied for a wireless EEG acquisition device to comfortably record EEG signals. Moreover, functional electrical stimulation can be realized by the device to facilitate post-stroke motor rehabilitation (Hsieh et al., 2022) (Figure 2A). Xu and co-workers developed a skin-like and integrated wearable sensor that can monitor a number of physiological human health indicators, including glucose, blood pressure, and caffeine. The highly integrated wearable conformal sensor was achieved by combining polymer composites and piezoelectric lead zirconate titanate ultrasound transducers (Sempionatto et al., 2021).

This section will discuss the design and applications of tissue-like deformable electronic skins. Electronic skins with stretchability and self-healing ability are highly desirable, especially for biointerfaces. For example, deformable electronic skins must conformally attach and mount to human skin and continuously collect the bio-signals. Deformable skin electronics from semiconductors to conductors, have been developed over the past few years (Trung and Lee, 2017).

### Stretchable materials-based electronics through geometric engineering

A geometric engineering approach is typically used to achieve stretchability in non-stretchable inorganic materials-based electronics. A structurally designed inorganic material can be configured with stretchability to interrogate, interface with, and modulate the skin after being supported by an elastomeric substrate (Chung et al., 2019; Yu et al., 2019). Planar 2D structures can be transformed geometrically into various extended 3D structures for more complex stretchability. Thin metal films are geometrically patterned to gain out-of-plane deformability in these 3D electronic designs (Miyamoto et al., 2017).

Using an ordered assembly strategy, Xue et al. could transform 2D thin films into sophisticated 3D structures on various curved surfaces (Xue Z. et al., 2022). Predetermined mechanical loadings deform elastomer substrates into flat/cylindrical configurations, followed by uniaxial/biaxial prestretching to drive buckling-guided assembly. By releasing predefined loadings, complex 3D structures are assembled in an orderly manner on curved substrates.

Moreover, Rogers and co-workers recently reported a set of hierarchical 3D framework assembly concepts. By leveraging multiple layers of prestretched elastomeric substrates, not only compressive buckling of 2D precursors bonded to them but also of themselves. Thus, 3D mesostructures mounted at multiple levels of 3D frameworks were created with complex, elaborate configurations (Zhao et al., 2022) (Figure 2B).

### Stretchable electronics with intrinsically stretchable materials

Geometrically engineered stretchable electronics or combinations of non-stretchable components and stretchable interconnects have disadvantages, such as high cost, fabrication complexity and low yield. Furthermore, the interconnections of wavy, wrinkled, buckled, serpentine, or net structures and the elastomeric composite are bulky, increasing the distance between unit devices. As a result, there are limitations to these approaches when it comes to fabricating high-density integrated circuits. To fabricate stretchable electronics with intrinsically stretchable components, new methods and fabrication technique have been developed. Intrinsically stretchable electronics can be achieved by the concept of “self-assembly enabled nanoconfinement” (Xu et al., 2017). Electronic skins based on polymer semiconductors with large stretchability were achieved utilizing the nanoconfinement effect, without compromising charge transport mobility. Jung and co-workers recently presented a nanocomposite comprised of Ag nanomaterials and block-copolymer elastomers for high-performance skin electronics (Jung et al., 2022). The nanocomposites are highly resistant to skin deformations (strains of 50%). Using staggered two-dimensional nanosheets with bond-free van der Waals (VDW) interfaces, Duan and colleagues designed van der Waals thin films (Yan et al., 2022). To ensure mechanical stretchability and malleability, these films feature bond-free VDW interfaces between the staggered 2D nanosheets that allow sliding and rotation degrees of freedom between neighboring nanosheets (Figure 2C).

The highly tunable properties of intrinsically stretchable polymer materials make them another category of tissue-like materials. Polymer composites with conductive inclusions are stretchable without complex patterning (Tee and Ouyang, 2018). Even after repeated mechanical deformations, stretchable polymer semiconductor films can retain electrical functionality through rational molecular-level design (Zheng et al., 2022). A printable and stretchable composite of viscoelastic, liquid-like polymer matrix has been recently reported. Ag flakes can spontaneously reorganize inside the matrix by cyclic mechanical stretching, leading to reconstructed microstructures and efficient conductive pathways (Wang et al., 2022e). With a pyramid-structured hydrogel and dielectric polymer, Jia et al. developed an electric double layer (EDL)-based soft stretchable self-powered pressure sensor (Jia et al., 2021). Wang and co-workers developed a highly stretchable polymer composite embedded with a three-dimensional liquid-metal network. The composite showed substantial increases in electromagnetic interference shielding effectiveness when stretched (Yao et al., 2020). The electrical conductivities of the 3D liquid-metal composite are also highly stretchable under large mechanical deformations. Liu et al. reported a printable conductor of biphasic Ga-In, with high stretchability (>1,000%), conductivity (2.06 × 106 S m^−1^), and negligible resistance change when strained (Liu et al., 2021). Their stretchable circuit board assemblies exhibited high performance for wearable sensing applications resulting from a scalable transfer-printing process.

### Self-healing materials for tissue-like deformable skin electronics

To achieve long-term stability, skin bioelectronics must be self-healing (Huang et al., 2015). Our bodies promote the natural healing of small wounds, such as scratches or cuts. A biomimetic healing ability in materials would allow microscopic and macroscopic injuries to be repaired. To realize intrinsic self-healing behaviors in synthetic materials, different molecular interactions have been explored (Terryn et al., 2021). Dynamic intermolecular interactions, such as dynamic covalent bonds as in Diels–Alder reactions, metal ion coordination, hydrogen bonding (H-bond), disulfide links, and π-π interaction, are used as the reversible bonding for intrinsic self-healing.

By combining weak hydrogen bonds and strong dipole-dipole interactions, Fu et al. designed a tough and efficient underwater self-healing supramolecular hydrogel (Fu et al., 2022). The hydrogel has high stretchability (up to 700%), toughness, and an almost 100% fast strain self-recovery. In the dynamic supramolecular hydrogel network, the hydrophobic effect of the acrylonitrile-acrylonitrile dipole-dipole interaction underwater removes the water molecules on the fractured interface. The H-bond then rebuilds between acrylamide and methacrylic acid, while dipole-dipole interactions at the interface facilitate underwater self-healing. Xue et al. recently reported on the design of a single-layer composite hydrogel with bulk capacitive junctions for mechanical sensors (Xue B. et al., 2022). The hydrogel network and graphene interact with strong yet dynamic interactions through peptide self-assembly. A hydrogel produced by this process can be stretched 77 times its original length and self-heal in a few minutes. Similar to the H-bonds, lithium bonds can also form ionic bonds because of the metallic nature of lithium. Using lithium bonds as depolymerization quenchers and dynamic mediators, Dang et al. produced high-performance ionoelastomers by melting lipoic acid (Dang et al., 2022). Self-healing hydrogels are also generated by forming dynamic aldehyde-amine crosslinks between chitosan and glutaraldehyde (Wang et al., 2022d).

A polymer gel system (PEGgel) based on a poly (hydroxyethyl methacrylate-co-acrylic acid) copolymerized in the presence of poly (ethylene glycol) (PEG) used as the liquid phase of the PEGgel was described by Wang Z. et al., 2022. PEG provides strong hydrogen bonding between the main chain ether and hydroxy groups when blended with polymers containing side hydroxy groups. Compared with the corresponding hydrogel or ethylene glycol-based gel, the PEGgel exhibits exceptional physical properties, such as high stretchability and rapid self-healing ability.

According to Su et al. (2022) a conducting polymer (poly (3,4-ethylenedioxythiophene): poly (styrenesulfonate), PEDOT:PSS) and a soft-polymer (poly (2-acrylamido-2-methyl-1-propanesulfonic acid, PAAMPSA) were combined to produce a stretchable and self-healable conducting film. When strain is applied or when severe damage occurs from stretching, the hydrogen and ionic bonds formed between PEDOT:PSS and PAAMPSA can undergo an energy dissipation mechanism through breakage of bonds. Under ambient conditions (≈23°C RT, ≈50% RH), these dynamic bonds can be reformed to restore the mechanical properties of the damaged area. The optimal film exhibits stretchability as high as 630% while possessing the self-healing ability for mechanical and electrical breakdowns.

## Conclusion and perspectives

In this mini-review, we highlighted recently reported advances in materials for next-generation wearable skin bioelectronics. These materials possess stretchability, self-healing ability and biocompatibility towards continuous, long-term health monitoring. While significant advancements have been made in this field, many practical challenges remain, including:

(i) The stretchability and biocompatibility of the materials mimics the properties of human skin. Soft-hard composites incorporated with tissue-like materials, combined with advanced manufacturing, are one solution to this issue (Lin et al., 2020).
(ii) The conductivity and sensitivity of the device, the ability to self-powering or wireless powering, and methods to integrate the technologies of all components (Nie et al., 2022).

Despite unmet challenges, advances in materials and soft devices have been a major driving force in the development of electronic skins (Cho et al., 2022). Materials scientists, device engineers, and doctors should collaborate closely to translate material-based bioelectronics to the clinic.
